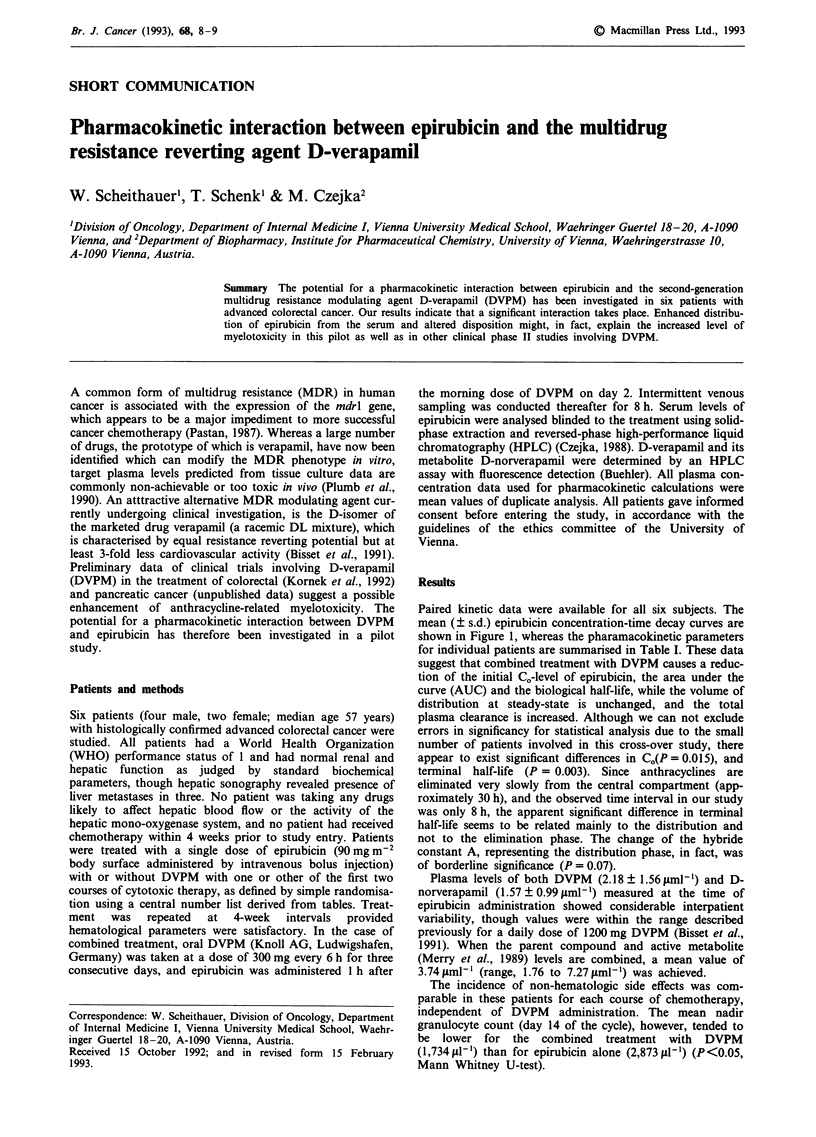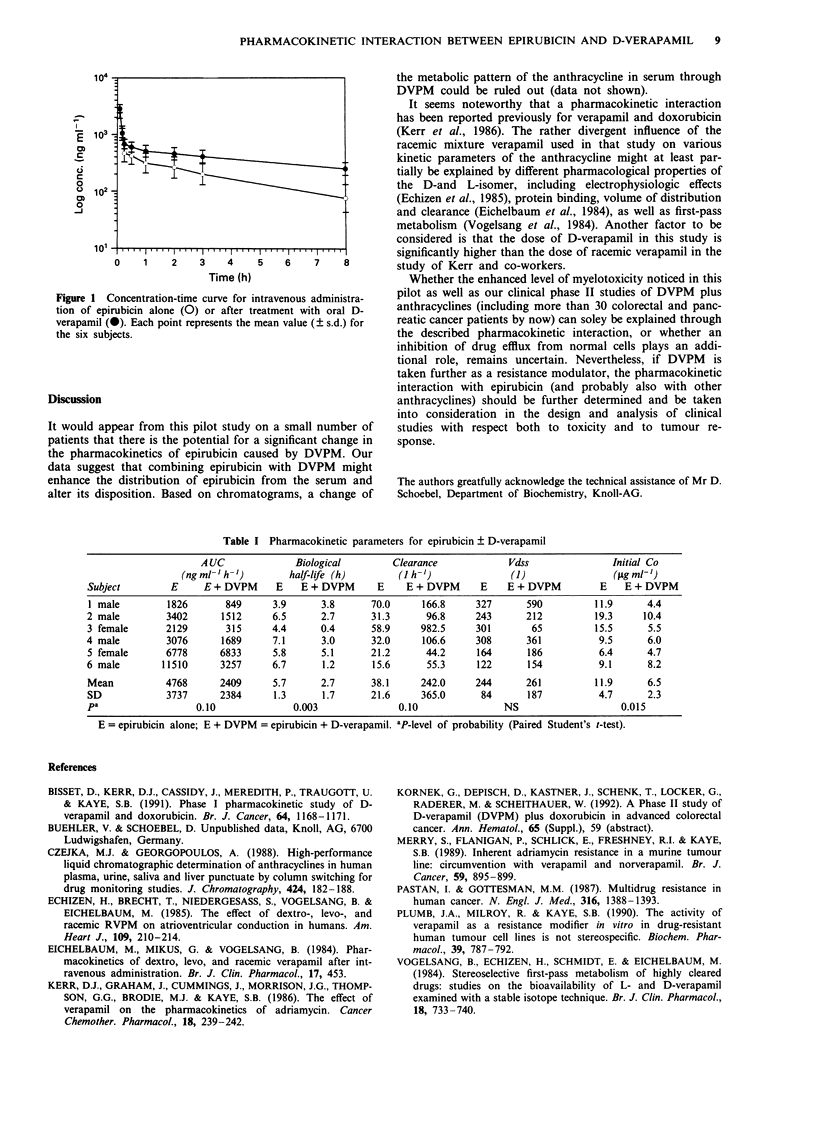# Pharmacokinetic interaction between epirubicin and the multidrug resistance reverting agent D-verapamil.

**DOI:** 10.1038/bjc.1993.277

**Published:** 1993-07

**Authors:** W. Scheithauer, T. Schenk, M. Czejka

**Affiliations:** Department of Internal Medicine I, Vienna University Medical School, Austria.

## Abstract

The potential for a pharmacokinetic interaction between epirubicin and the second-generation multidrug resistance modulating agent D-verapamil (DVPM) has been investigated in six patients with advanced colorectal cancer. Our results indicate that a significant interaction takes place. Enhanced distribution of epirubicin from the serum and altered disposition might, in fact, explain the increased level of myelotoxicity in this pilot as well as in other clinical phase II studies involving DVPM.


 © Macmillan Press Ltd., 1993

SHORT COMMUNICATION

### Pharmacokinetic interaction between epirubicin and the multidrug resistance reverting agent D-verapamil

W. Scheithauer[1], T. Schenk[1] & M. Czejka[2]

[1]*Division of Oncology, Department of Internal Medicine I, Vienna University Medical School, Waehringer Guertel 18–20, A-1090 Vienna, and* [2]*Department of Biopharmacy, Institute for Pharmaceutical Chemistry, University of Vienna, Waehringerstrasse 10, A-1090 Vienna, Austria.*

**Summary** The potential for a pharmacokinetic interaction between epirubicin and the second-generation multidrug resistance modulating agent D-verapamil (DVPM) has been investigated in six patients with advanced colorectal cancer. Our results indicate that a significant interaction takes place. Enhanced distribution of epirubicin from the serum and altered disposition might, in fact, explain the increased level of myelotoxicity in this pilot as well as in other clinical phase II studies involving DVPM.

A common form of multidrug resistance (MDR) in human cancer is associated with the expression of the *mdr*1 gene, which appears to be a major impediment to more successful cancer chemotherapy (Pastan, 1987). Whereas a large number of drugs, the prototype of which is verapamil, have now been identified which can modify the MDR phenotype *in vitro*, target plasma levels predicted from tissue culture data are commonly non-achievable or too toxic *in vivo* (Plumb *et al.*, 1990). An atttractive alternative MDR modulating agent currently undergoing clinical investigation, is the D-isomer of the marketed drug verapamil (a racemic DL mixture), which is characterised by equal resistance reverting potential but at least 3-fold less cardiovascular activity (Bisset *et al.*, 1991). Preliminary data of clinical trials involving D-verapamil (DVPM) in the treatment of colorectal (Kornek *et al.*, 1992) and pancreatic cancer (unpublished data) suggest a possible enhancement of anthracycline-related myelotoxicity. The potential for a pharmacokinetic interaction between DVPM and epirubicin has therefore been investigated in a pilot study.

### Patients and methods

Six patients (four male, two female; median age 57 years) with histologically confirmed advanced colorectal cancer were studied. All patients had a World Health Organization (WHO) performance status of 1 and had normal renal and hepatic function as judged by standard biochemical parameters, though hepatic sonography revealed presence of liver metastases in three. No patient was taking any drugs likely to affect hepatic blood flow or the activity of the hepatic mono-oxygenase system, and no patient had received chemotherapy within 4 weeks prior to study entry. Patients were treated with a single dose of epirubicin (90 mg $m^{-2}$ body surface administered by intravenous bolus injection) with or without DVPM with one or other of the first two courses of cytotoxic therapy, as defined by simple randomisation using a central number list derived from tables. Treatment was repeated at 4-week intervals provided hematological parameters were satisfactory. In the case of combined treatment, oral DVPM (Knoll AG, Ludwigshafen, Germany) was taken at a dose of 300 mg every 6 h for three consecutive days, and epirubicin was administered 1 h after the morning dose of DVPM on day 2. Intermittent venous sampling was conducted thereafter for 8 h. Serum levels of epirubicin were analysed blinded to the treatment using solid-phase extraction and reversed-phase high-performance liquid chromatography (HPLC) (Czejka, 1988). D-verapamil and its metabolite D-norverapamil were determined by an HPLC assay with fluorescence detection (Buehler). All plasma concentration data used for pharmacokinetic calculations were mean values of duplicate analysis. All patients gave informed consent before entering the study, in accordance with the guidelines of the ethics committee of the University of Vienna.

Correspondence: W. Scheithauer, Division of Oncology, Department of Internal Medicine I, Vienna University Medical School, Waehringer Guertel 18–20, A-1090 Vienna, Austria.
Received 15 October 1992; and in revised form 15 February 1993.

### Results

Paired kinetic data were available for all six subjects. The mean (± s.d.) epirubicin concentration-time decay curves are shown in Figure 1, whereas the pharamacokinetic parameters for individual patients are summarised in Table I. These data suggest that combined treatment with DVPM causes a reduction of the initial $C_0$-level of epirubicin, the area under the curve (AUC) and the biological half-life, while the volume of distribution at steady-state is unchanged, and the total plasma clearance is increased. Although we can not exclude errors in significancy for statistical analysis due to the small number of patients involved in this cross-over study, there appear to exist significant differences in $C_0(P = 0.015)$, and terminal half-life ($P = 0.003$). Since anthracyclines are eliminated very slowly from the central compartment (approximately 30 h), and the observed time interval in our study was only 8 h, the apparent significant difference in terminal half-life seems to be related mainly to the distribution and not to the elimination phase. The change of the hybride constant A, representing the distribution phase, in fact, was of borderline significance ($P = 0.07$).

Plasma levels of both DVPM (2.18 ± 1.56 $\mu$m$l^{-1}$) and D-norverapamil (1.57 ± 0.99 $\mu$m$l^{-1}$) measured at the time of epirubicin administration showed considerable interpatient variability, though values were within the range described previously for a daily dose of 1200 mg DVPM (Bisset *et al.*, 1991). When the parent compound and active metabolite (Merry *et al.*, 1989) levels are combined, a mean value of 3.74 $\mu$m$l^{-1}$ (range, 1.76 to 7.27 $\mu$m$l^{-1}$) was achieved.

The incidence of non-hematologic side effects was comparable in these patients for each course of chemotherapy, independent of DVPM administration. The mean nadir granulocyte count (day 14 of the cycle), however, tended to be lower for the combined treatment with DVPM (1,734 $\mu l^{-1}$) than for epirubicin alone (2,873 $\mu l^{-1}$) ($P<0.05$, Mann Whitney U-test).

**Figure 1** Concentration-time curve for intravenous administration of epirubicin alone (○) or after treatment with oral D-verapamil (●). Each point represents the mean value (± s.d.) for the six subjects.

### Discussion

It would appear from this pilot study on a small number of patients that there is the potential for a significant change in the pharmacokinetics of epirubicin caused by DVPM. Our data suggest that combining epirubicin with DVPM might enhance the distribution of epirubicin from the serum and alter its disposition. Based on chromatograms, a change of the metabolic pattern of the anthracycline in serum through DVPM could be ruled out (data not shown).

It seems noteworthy that a pharmacokinetic interaction has been reported previously for verapamil and doxorubicin (Kerr *et al.*, 1986). The rather divergent influence of the racemic mixture verapamil used in that study on various kinetic parameters of the anthracycline might at least partially be explained by different pharmacological properties of the D-and L-isomer, including electrophysiologic effects (Echizen *et al.*, 1985), protein binding, volume of distribution and clearance (Eichelbaum *et al.*, 1984), as well as first-pass metabolism (Vogelsang *et al.*, 1984). Another factor to be considered is that the dose of D-verapamil in this study is significantly higher than the dose of racemic verapamil in the study of Kerr and co-workers.

Whether the enhanced level of myelotoxicity noticed in this pilot as well as our clinical phase II studies of DVPM plus anthracyclines (including more than 30 colorectal and pancreatic cancer patients by now) can soley be explained through the described pharmacokinetic interaction, or whether an inhibition of drug efflux from normal cells plays an additional role, remains uncertain. Nevertheless, if DVPM is taken further as a resistance modulator, the pharmacokinetic interaction with epirubicin (and probably also with other anthracyclines) should be further determined and be taken into consideration in the design and analysis of clinical studies with respect both to toxicity and to tumour response.

The authors greatfully acknowledge the technical assistance of Mr D. Schoebel, Department of Biochemistry, Knoll-AG.

**Table I** Pharmacokinetic parameters for epirubicin ± D-verapamil

| Subject | *AUC* (*ng ml⁻¹ h⁻¹*) *E* | *E* + DVPM | *Biological half-life (h)* E | E + DVPM | *Clearance* (*l h⁻¹*) E | E + DVPM | *Vdss* (*l*) E | E + DVPM | *Initial Co* (*µg ml⁻¹*) E | E + DVPM |
|---|---|---|---|---|---|---|---|---|---|---|
| 1 male | 1826 | 849 | 3.9 | 3.8 | 70.0 | 166.8 | 327 | 590 | 11.9 | 4.4 |
| 2 male | 3402 | 1512 | 6.5 | 2.7 | 31.3 | 96.8 | 243 | 212 | 19.3 | 10.4 |
| 3 female | 2129 | 315 | 4.4 | 0.4 | 58.9 | 982.5 | 301 | 65 | 15.5 | 5.5 |
| 4 male | 3076 | 1689 | 7.1 | 3.0 | 32.0 | 106.6 | 308 | 361 | 9.5 | 6.0 |
| 5 female | 6778 | 6833 | 5.8 | 5.1 | 21.2 | 44.2 | 164 | 186 | 6.4 | 4.7 |
| 6 male | 11510 | 3257 | 6.7 | 1.2 | 15.6 | 55.3 | 122 | 154 | 9.1 | 8.2 |
| Mean | 4768 | 2409 | 5.7 | 2.7 | 38.1 | 242.0 | 244 | 261 | 11.9 | 6.5 |
| SD | 3737 | 2384 | 1.3 | 1.7 | 21.6 | 365.0 | 84 | 187 | 4.7 | 2.3 |
| $P^a$ | 0.10 | | 0.003 | | 0.10 | | NS | | 0.015 | |

E = epirubicin alone; E + DVPM = epirubicin + D-verapamil. [a]*P*-level of probability (Paired Student's *t*-test).

### References

BISSET, D., KERR, D.J., CASSIDY, J., MEREDITH, P., TRAUGOTT, U. & KAYE, S.B. (1991). Phase I pharmacokinetic study of D-verapamil and doxorubicin. *Br. J. Cancer*, **64**, 1168–1171.

BUEHLER, V. & SCHOEBEL, D. Unpublished data, Knoll, AG, 6700 Ludwigshafen, Germany.

CZEJKA, M.J. & GEORGOPOULOS, A. (1988). High-performance liquid chromatographic determination of anthracyclines in human plasma, urine, saliva and liver punctuate by column switching for drug monitoring studies. *J. Chromatography*, **424**, 182–188.

ECHIZEN, H., BRECHT, T., NIEDERGESASS, S., VOGELSANG, B. & EICHELBAUM, M. (1985). The effect of dextro-, levo-, and racemic RVPM on atrioventricular conduction in humans. *Am. Heart J.*, **109**, 210–214.

EICHELBAUM, M., MIKUS, G. & VOGELSANG, B. (1984). Pharmacokinetics of dextro, levo, and racemic verapamil after intravenous administration. *Br. J. Clin. Pharmacol.*, **17**, 453.

KERR, D.J., GRAHAM, J., CUMMINGS, J., MORRISON, J.G., THOMPSON, G.G., BRODIE, M.J. & KAYE, S.B. (1986). The effect of verapamil on the pharmacokinetics of adriamycin. *Cancer Chemother. Pharmacol.*, **18**, 239–242.

KORNEK, G., DEPISCH, D., KASTNER, J., SCHENK, T., LOCKER, G., RADERER, M. & SCHEITHAUER, W. (1992). A Phase II study of D-verapamil (DVPM) plus doxorubicin in advanced colorectal cancer. *Ann. Hematol.*, **65** (Suppl.), 59 (abstract).

MERRY, S., FLANIGAN, P., SCHLICK, E., FRESHNEY, R.I. & KAYE, S.B. (1989). Inherent adriamycin resistance in a murine tumour line: circumvention with verapamil and norverapamil. *Br. J. Cancer*, **59**, 895–899.

PASTAN, I. & GOTTESMAN, M.M. (1987). Multidrug resistance in human cancer. *N. Engl. J. Med.*, **316**, 1388–1393.

PLUMB, J.A., MILROY, R. & KAYE, S.B. (1990). The activity of verapamil as a resistance modifier *in vitro* in drug-resistant human tumour cell lines is not stereospecific. *Biochem. Pharmacol.*, **39**, 787–792.

VOGELSANG, B., ECHIZEN, H., SCHMIDT, E. & EICHELBAUM, M. (1984). Stereoselective first-pass metabolism of highly cleared drugs: studies on the bioavailability of L- and D-verapamil examined with a stable isotope technique. *Br. J. Clin. Pharmacol.*, **18**, 733–740.